# Smart microscopy: adaptive microscope control to improve the way we see life

**DOI:** 10.1038/s44303-026-00145-y

**Published:** 2026-03-03

**Authors:** Alfredo Rates, Josiah B. Passmore, Nils Norlin, Lukas C. Kapitein

**Affiliations:** 1 Cell Biology, Neurobiology and Biophysics, Department of Biology, Utrecht University, Utrecht, The Netherlands; 2 Department of Experimental Medical Science, Lund University Bioimaging Centre (LBIC) & NanoLund, Lund University, Lund, Sweden

**Keywords:** Biological techniques, Biophysics, Cell biology, Engineering, Optics and photonics

## Abstract

Smart microscopy lies at the intersection of biology, optics, engineering, and computer science. Unlike traditional microscopes, smart systems actively adapt their acquisition settings in real time based on information extracted from the sample, allowing experiments to navigate competing demands such as resolution, speed and sample health. In this review, we present a practical framework for what makes a microscope “smart,” defining smart microscopy as the combination of real-time analysis, feedback control, and automated actuation. To guide implementation, we classify smart microscopy approaches by experimental goal (quality-, event-, target-, information- or outcome-driven) and discuss the corresponding strategies for analysis and control. Finally, we highlight key challenges and the growing role of community-driven efforts in making smart microscopy more accessible and widely adopted across the life sciences.

## Why smart?

Over the years, microscopes have advanced dramatically, driven by progress in electronics, optics, and mechanics. However, these improvements face fundamental physical constraints, such as the diffraction limit, or the limited photon budget of many fluorophores. In the life sciences, imaging is further limited not just by the microscope’s design, but also by how the imaging process affects the sample itself^1^. These combined constraints and necessary trade-offs to be made are often illustrated by the so-called “pyramid of frustration”^2–4^, which highlights the trade-offs between spatial resolution, imaging speed, signal-to-noise ratio (SNR), field of view, and sample viability.

The pyramid of frustration captures the inherent compromises when imaging biological samples: achieving high spatial resolution typically comes at the expense of imaging speed or may damage sensitive biological samples, while preserving sample integrity often means sacrificing resolution or acquisition speed. As a result, researchers must constantly balance these competing demands depending on the scientific question. These inherent trade-offs motivate the development of adaptive solutions to balance competing priorities.

Microscopes are employed in a diverse set of experimental contexts, sometimes even with conflicting requirements. Some applications (e.g., large-scale tissue mapping or whole-organ imaging) demand large areas and volumes to be covered. In contrast, other applications (e.g., live-cell imaging or developmental biology studies) require gentle imaging conditions with high temporal and spatial resolution. Although modern microscopes often meet each technical demand in isolation, they cannot be combined in a single experiment. This challenge is nowadays addressed with microscope automation, adapting acquisition strategies for specific and complex experiments, thereby unlocking the full potential of existing systems^5,6^, and opening the possibility for multi-scale microscopy imaging^7^.

Smart microscopy is thus the automation of microscopes such that they make decisions on the fly to change acquisition settings, based on information from the sample under study, to optimize data collection for the given experiment. With such automation, an imaging session has the potential to include multiple imaging modalities, even when each modality has different or opposite optimization criteria^8,9^. As an example, let’s consider the observation of the cell cycle in 2D cultured cells. Mitosis is a rare but fast-paced event that requires high temporal resolution to be captured accurately. However, maintaining high temporal resolution throughout the entire cell cycle would result in excessive phototoxicity and photobleaching. To address this, a smart microscope can begin by imaging at a lower temporal resolution and then dynamically switch to a higher temporal resolution only when mitotic events are detected. Furthermore, the microscope can adapt the field of view in real time for multi-scale imaging such that only the cell undergoing the targeted stage is observed. In this context, *Image acquisition* encapsulates the whole process from illumination of the sample to saving the data, including all steps in between such as illumination strategy, stage movement, environment control, and on-the-fly data analysis.

**Fig. 1:**
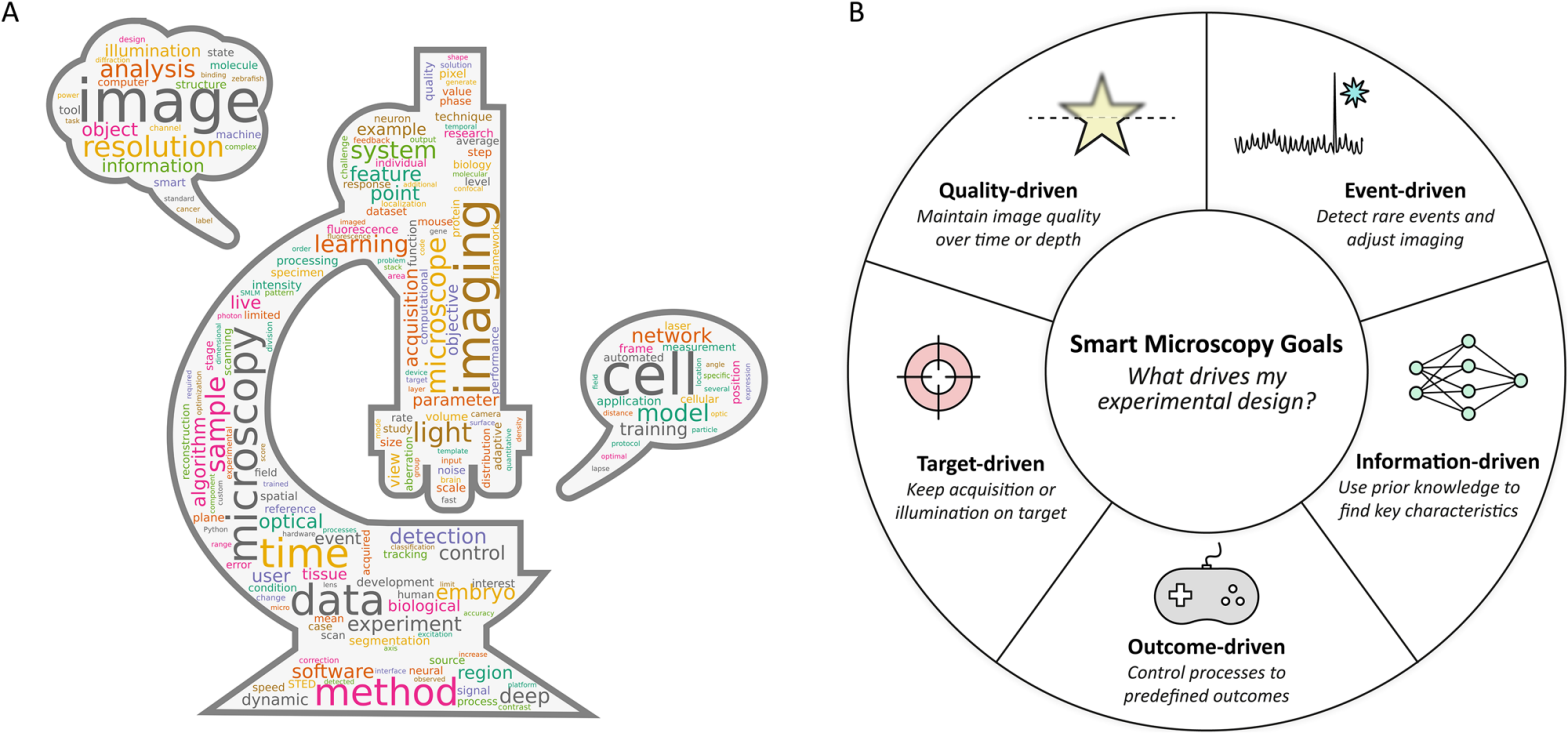
Classification of smart microscopy and its presence in the literature. **A** A word cloud from the full text of the >200 references cited in this review, highlighting the diversity within smart microscopy applications. **B** An illustration of the motives (smart microscopy goals) that drive the experimental design of a smart microscopy experiment.

Smart microscopy enables imaging of highly dynamic or complex biological processes that are difficult to capture manually^10,11^, such as rapid cellular events or large-scale tissue dynamics. But smart microscopy is not limited to ultrafast, complex experiments. Although some experiments are in principle possible to carry out manually, automation increases precision, potentially augments statistical value, and alleviates the burden of such experiments to the user. Thus, smart microscopy offers versatility across many biological applications (Fig. 1A) and can be tailored to diverse imaging needs^12^.

In this review, we focus on light microscopy in the life sciences. Still, feedback in imaging (and by extension smart microscopy) has appeared useful in many different imaging modalities, beyond light microscopy. Automation and feedback loops can be found in imaging modalities such as electron microscopy^13–18^ and atomic force microscopy^19,20^, and outside of life science such as in metrology^21,22^ and astronomy^23,24^.

## Beginnings of smart microscopy

The development of the microscope can be traced back to the late 1600s^25–30^. Since its invention, the microscope has undergone remarkable evolution, driven by advances in optics, controllable light sources, high-precision mechanical control, and novel detectors. These technological and engineering breakthroughs have significantly enhanced both the design and performance of modern microscopes. Beyond developments in engineering and optics, light microscopy had a crucial change thanks to the development of fluorescent labels, in particular the introduction of the green fluorescent protein to enable straightforward live-cell imaging of specific targets^31,32^.

Automation entered microscopy in the late 20th century, with the first commercial microscopes using motorized stages appearing in the 1970s, such as the Zeiss Axiomat. Later, in the 1980s, the first microscopes with an integrated digital camera appeared in Japan. Early digital microscope models include the Zeiss Axiovert 135 and the Leica M40. The same decade, Nikon developed their autofocus system to automatically find the position of the sample carrier along the z-axis. This functionality appeared first in the Optiphot 2 and Diaphot TMD series. These automated systems did have an automatic observer and actuator^33,34^, but because the automation happens in parallel to the imaging and does not affect the acquisition parameters, we do not yet consider these systems smart microscopy.

As soon as the automated microscope arrived, ideas about smarter microscopes started to emerge^34–36^. First implementations of on-the-fly image analysis^37^, ideas towards single-cell segmentation^38,39^ and even custom-built analog smart instruments^40^ predate commercial microscopes with integrated cameras and motorized stages. As the field progressed, innovations in image processing, sample handling, and sample identification enhanced the capabilities of automated microscopy. Computer-controlled microscopes introduced automated feedback, significantly enhancing performance in many applications.

At the end of the 20th century and beginning of the 21st, there was a rapid improvement in digital technologies. These advancements also influenced microscopy, by increasing the computational power available for data processing, improving sensitivity and dynamic range of cameras and sensors, enhancing precision in detection and motorized control of mechanical devices.

Advances in microscopy technology coincided with growing use of fluorescent proteins for live imaging, expanding the range of observable biological processes^41,42^. Fluorescence live imaging allowed for breakthroughs in life sciences, but also brought new challenges, namely sample health and bleaching. Soon, imaging was not only optimized to enhance the resulting image but also to make sure the sample was treated gently^43^. With that, the microscope automation was no longer detached from sample preservation.

Although many improvements in technology were developed by industry, the need for tailor-made solutions pushed scientists in academia to complement commercial models with in-house made devices. Thanks to global standardization in designs and the emergence of 3D printing, along with the development of open-source microcontrollers such as Arduino and Raspberry-Pi, there was a democratization of automation. Furthermore, in 2005 the microscope community saw the release of the microscope controller software MicroManager^44^. While tools such as MetaMorph^45^ and custom-built LabVIEW microscope controllers were available, MicroManager became widely adopted due to its open-source, microscope-agnostic design. MicroManager aimed to standardize microscope control and allowed for automation in a somewhat universal way. More recently, more tools and platforms for microscope control have been developed, such as ImSwitch^46,47^, Navigate^48^, MicroMator^49^, Micropilot^50^, Microscope-Cockpit^51^, Arkitekt^52^. By synchronizing multiple components, such as light sources and imaging stages, microscopy automation allows for real-time adjustments, enabling high-throughput data acquisition and the study of short- and long-term dynamic phenomena with unprecedented precision and consistency. As these additions allowed for true real-time feedback on the biological sample, we consider that at this point microscopy entered a new era - smart microscopy. Nowadays, most smart microscopy studies that are published use custom solutions for microscope automation^53,54^, either building on top of existing platforms or from the ground up. Current smart microscopy solutions still need to show viability for mass adoption and often lack key automation factors such as parallelization, modularity, and user-friendliness. But the fast pace of new developments show a promising future for the field.

In parallel, computer science is undergoing a revolutionary transformation powered by artificial intelligence (AI). By enabling computers to independently perform tasks that were previously only possible to be done by a human, AI has fundamentally changed how scientists approach complex problems. This development has been particularly pronounced in image analysis, where AI improvements have both accelerated and been accelerated by advances in visual processing capabilities. Today, AI models and tools are widely used for image analysis across science, industry, and even in everyday life. Unsurprisingly, AI has also had a profound impact on the field of microscopy. While the definition of AI can be debated, we employ the term AI mainly to describe deep learning algorithms, based on large neuronal networks. Many globally used tools such as image segmentation and large language models (LLMs) are at their core based on deep learning, and because users are familiar with such tools, we refer to them as AI. This does not mean, however, that machine learning solutions for classification or image analysis have no relevance for smart microscopy experiments.

AI was first introduced to the microscopy field for image analysis, data extraction, and image enhancement^10,55,56^. As AI methods advanced, they were increasingly applied to end-to-end analysis and experimental design^57,58^. The rise of LLMs such as chatGPT has made many microscope users wonder, could such LLMs be integrated into the microscope?^59,60^ How can imaging automation be improved with AI?^61^ And thus, with new improvements in microscope hardware, the democratization of automation, and the development of AI tools, microscopes began to evolve from passive elements of life science experiments to active, dynamic components^62^.

## Smart microscopy goals

Recent literature highlights the growing application space for smart microscopy, and also showcases the development of versatile tools designed to enable it^63^. Many smart microscopy software solutions are actively being developed for different imaging modalities and experiment paradigms. However, even though some solutions can be considered more generalizable than others, adapting them to new workflows or biological systems often remains a non-trivial challenge.

The first questions to consider when applying smart microscopy are: what motivates my experiment? What drives automation? Is the goal, for example, to follow a moving target, to dynamically change the resolution based on a rare event, or to keep an object of interest in focus through a changing environment? To guide this decision and differentiate smart microscopy tools, we here consider a classification system based on the goals of smart microscopy. We classify goals in five categories: Quality-driven, Event-driven, Target-driven, Information-driven, and Outcome-driven. The categories are not mutually exclusive and a given smart microscopy approach may belong to multiple categories. These categories are illustrated in Fig. 1B. This is built upon the classification system we presented in the community-driven white paper as part of the euro-bioImaging smart microscopy working group (SMWG)^63^.

### Quality driven

When the goal is to maintain the quality of our images in a dynamic experiment, consider ‘quality-driven’ smart microscopy. Here, *quality* refers to an abstract, experiment-specific metric. Quality can refer to SNR, level of optical aberrations, or desired sharpness, but also photobleaching minimization, illumination plane optimization, or measurement synchronization.

An illustrative example of quality-driven microscopy is the use of adaptive optics. Adaptive optics uses wavefront correction optics such as deformable mirrors to correct for aberrations. This correction can be done to the incident light to achieve an optimal illumination profile and point spread function, or to the fluorescence or transmitted light to correct aberrations at the detector plane. Maintaining the quality of the illumination pattern is of particular importance for techniques such as single molecule localization microscopy in deep tissue^64,65^.

**Fig. 2:**
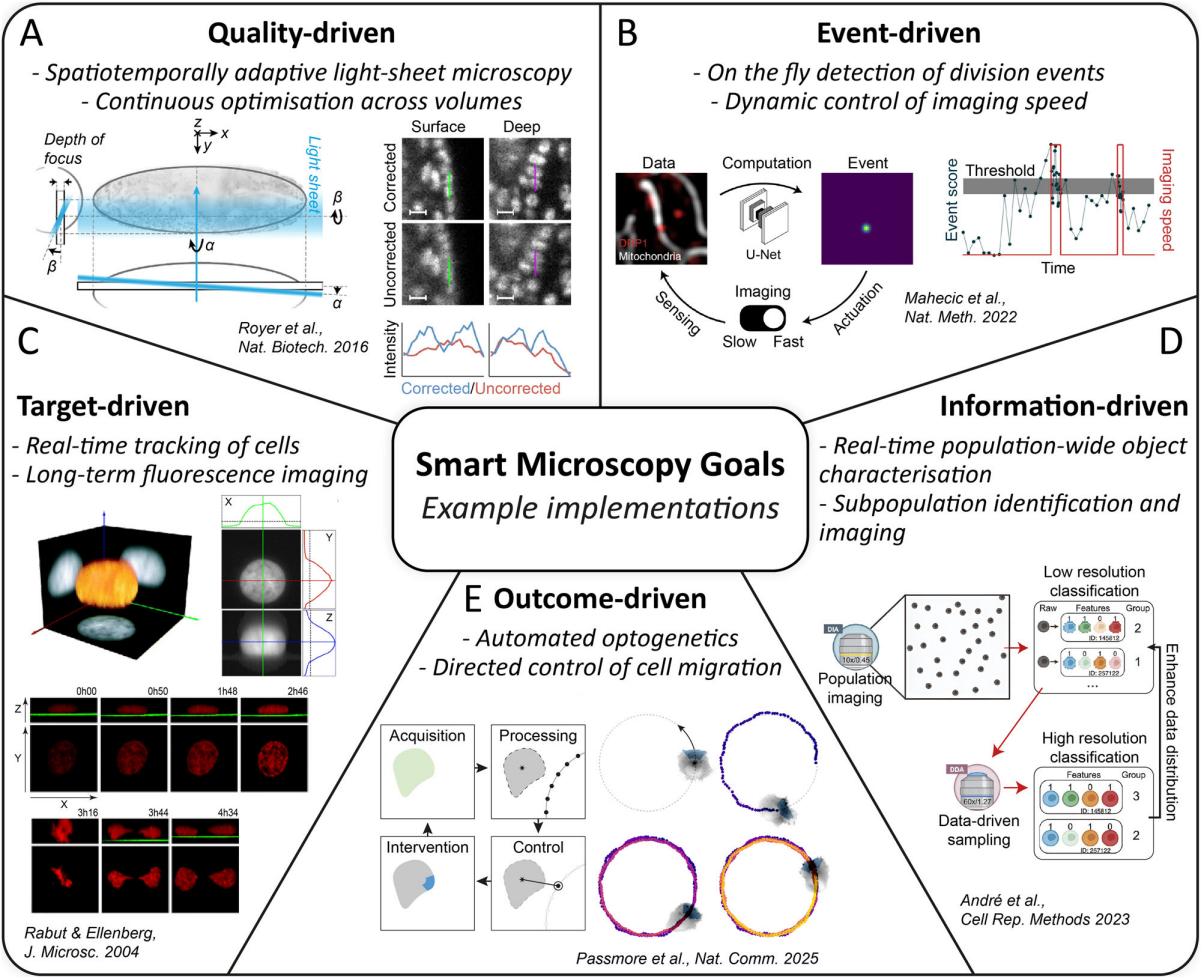
Example implementations of smart microscopy in the literature classified by the set-out goals of the experiment. **A** A quality-driven approach from Royer et al. to align the illumination and imaging plane of a light-sheet microscope on the fly. Reprinted by permission as indicated in the Terms and Conditions licenced by Springer Nature. **B** Event-driven microscopy from Mahecic et al. used to detect and thus image mitochondria division. Reprinted by permission as indicated in the Terms and Conditions licenced by Springer Nature. **C** Target-driven approach by Rabut & Ellenberg for single-cell tracking in 3D. Reprinted by permission as indicated in the Terms and Conditions licenced by Wiley Company. **D** Example of information-driven microscopy from André et al. to identify, classify and image different phenotypes based on population-level information. **E** Outcome-driven microscope from Passmore et al. that uses optogenetics for cell guidance with adaptive illumination patterns.

There are many examples of quality-driven smart microscopy in the literature. Among them, quality-driven microscopy has been used to enhance the quality of light-sheet microscopy^66–68^, to correct for aberrations in fluorescence (scanning) microscopy^69–72^, focus correction^73,74^, adaptive multirate^75^, and photon budget optimization^76–79^. The results from Royer et al.^80^ exemplify quality-driven microscopy in more detail, where they applied on-the-fly adaptive illumination in light-sheet microscopy to correct for heterogeneity and movement in living organisms during imaging (see Fig. 2A). The main goal is to ensure the illumination plane and the imaging plane are constantly aligned. But due to aberrations, heterogeneity, and movement, such correction has to be done on the fly. They introduced the AutoPilot platform to do so, which controls key parameters of the light sheet microscope, such as position and angle of both the illumination and detection angle.

### Event driven

If the goal is to detect rare events, “event-driven” smart microscopy is used. Usually, a rare event triggers a change in the acquisition settings, either to capture the event with high spatiotemporal resolution or to signal the end of the experiment or the beginning of a new imaging cycle. A clear example of a rare event is cell division; if the aim is to study cell division with high resolution over many cycles, event-driven microscopy can be used to observe changes in cell morphology prior to division with low fidelity, and start the high-resolution acquisition only at relevant time frames.

Although an event is generally a temporal phenomenon, event-driven smart microscopy can also be used when scanning a large static sample. In this case, a static event (e.g., specimen with a specific phenotype) is found dynamically while capturing the whole sample. A smart sample finder would be an implementation of this type of event-driven microscopy.

Event-driven microscopy has been used to detect cell signaling^81–83^, cell division^53,84,85^, protein aggregation^86^, chromatin unfolding^87^, secretion^88^, infection^88^, RNA-protein condensate^89^, membrane dynamics^90^, and single-molecule blinking^91^. Many implementations of event-driven microscopy include multi-modal or multi-scale imaging, using the event to trigger a change of scale (magnification) or modality (technique). An illustrative example of event-driven microscopy is the publication from Mahecic et al.^92^, who used smart microscopy to recognize mitochondrial divisions (see Fig. 2B). In the study, the researchers imaged mitochondria inside COS7 cells and detected mitochondria division events using a neuronal network. They start by imaging with long time intervals to minimize light exposure and thus phototoxicity, and once the event is detected, switch to short time intervals to observe the dynamics of mitochondria division.

### Target driven

“Target-driven” smart microscopy is used to ensure that the imaged or illuminated region is consistently on-target throughout the experiment. The target can be, for example, the position of a specimen, to ensure it is constantly centered, or the rotation of the sample in a capillary, to maintain a consistent imaging region. This is particularly relevant for techniques such as photoactivation, FRAP, or open-loop optogenetics, where constant illumination in a specific region of the sample is essential to activate or deactivate particular biological processes over time. Target-driven microscopy can also be used in combination with multi-scale imaging to image at the (sub-)cellular while maintaining the population level at a steady state.

Demonstrations of target-driven microscopy include cell and molecule tracking^93–95^, stable optogenetic illumination for cell migration^96^, STED power modulation^97^, phenotype screening^98^, and long-term whole organism tracking^54,99,100^. An early example of target-driven microscopy is the research from Rabut & Ellenberg^93^, who used 2D intensity projections to track single cells in 3D in a fluorescent microscope (see Fig. 2C). Using NRK cells tagged with DiHcRed, they image z-stacks of a single cell and then project the volumetric data into the axes XY, YZ, and ZX. With that, they obtained a 3D coordinate of the centroid of the cell. This was used to track the cell, with successful results even during cell division. Thanks to this automation, they were able to obtain 20 times the amount of data they were previously measuring.

### Information driven

In many cases, a significant amount of prior information about the sample under study already exists. This is especially true when studying model systems, such as yeast, zebrafish, and C. elegans. In such cases, “information-driven” smart microscopy proves useful. The goal of information-driven microscopy is to leverage prior knowledge to find key characteristics in the sample. Thus, the microscope image the sample in search for new information, avoiding portions that have only redundant information.

Information-driven microscopy is not only limited to prior information but can also include population information, e.g., when multiple specimens are being observed simultaneously. Then, the information of one specimen or an ensemble of specimens, can be used to optimally guide further microscope acquisitions.

Information-driven microscopy has been used in zebrafish to find regions of interest^101^ or perform robotic surgery^102^, and also to predict and calibrate yeast cell quantity^103^. A representative example is the publication from André et al.^104^, who used population-wide data for phenotype classification and high-resolution multi-modal microscope imaging (see Fig. 2D). In particular, they image a population of HeLa cells with low magnification. From this low resolution image, population wide features were extracted allowing the identification of outliers. This approach was used to discriminate between non expressing vs expressing cells in a transfection assay, and non infected cells and infected cells following bacterial infection. With this information, target cells could be identified and imaged in high resolution.

### Outcome driven

To move forward from observing a sample to actively interacting with it, “outcome-driven” smart microscopy can be used. This approach uses the biological response of the specimen to dynamically adjust the microscope’s acquisition settings, creating a real-time feedback loop between the hardware and the biological system.

To achieve outcome-driven microscopy, a system is needed that introduces controlled input signals, influencing biological processes and resulting in observable effects. There are many such signals that can be used to induce changes in biology^105^. This could be light excitation for bleaching or optogenetics^106–111^, temperature of an incubator or local infrared light^112–114^, inflow of resources or drugs^115,116^, or strong magnetic fields or electric current^117–119^, to name a few. Notably, optogenetics has emerged as the most widely used technique for observing induced biological changes. As a result, most outcome-driven studies rely on optogenetics. The use of external controllable signals is a necessary, but not sufficient condition for outcome driven microscopy. An external signal is needed to control the biological system, but needs to be used to control the system on the fly, driving it to the desired state.

Outcome-driven smart microscopy has a large overlap with Cybergenetics^120–125^. Cybergenetics is the combination of control theory and biology by the use of optogenetics, specifically^126^. Cybergenetics can be used to control single cells and cell populations, but also expands beyond the boundaries of outcome-driven microscopy, including synthetic biology to encode control systems within a living cell.

Outcome-driven microscopy has been implemented to control neuronal activity of C. elegans^127,128^, cell transcription regulation^129^, cell morphology^130^, and cell differentiation^131^. An example of this classification is shown by Passmore et al.^132^, implementing outcome-driven microscopy to optogenetically induce migration in HT1080 cells to follow a predefined path (see Fig. 2E). In such a case, the signal induced in the system is the local blue-light illumination, which changes the position of the cell. And because of induced cell movement, the illumination pattern and field-of-view has to be adapted, creating the characteristic feedback loop of outcome-driven smart microscopy.

## Smart microscopy strategies

After questioning what motivates the smart microscopy experiment, it is also important to consider how automation can be used to reach the smart microscopy goal. In other words: what does this automation adjust on the microscope?

**Fig. 3:**
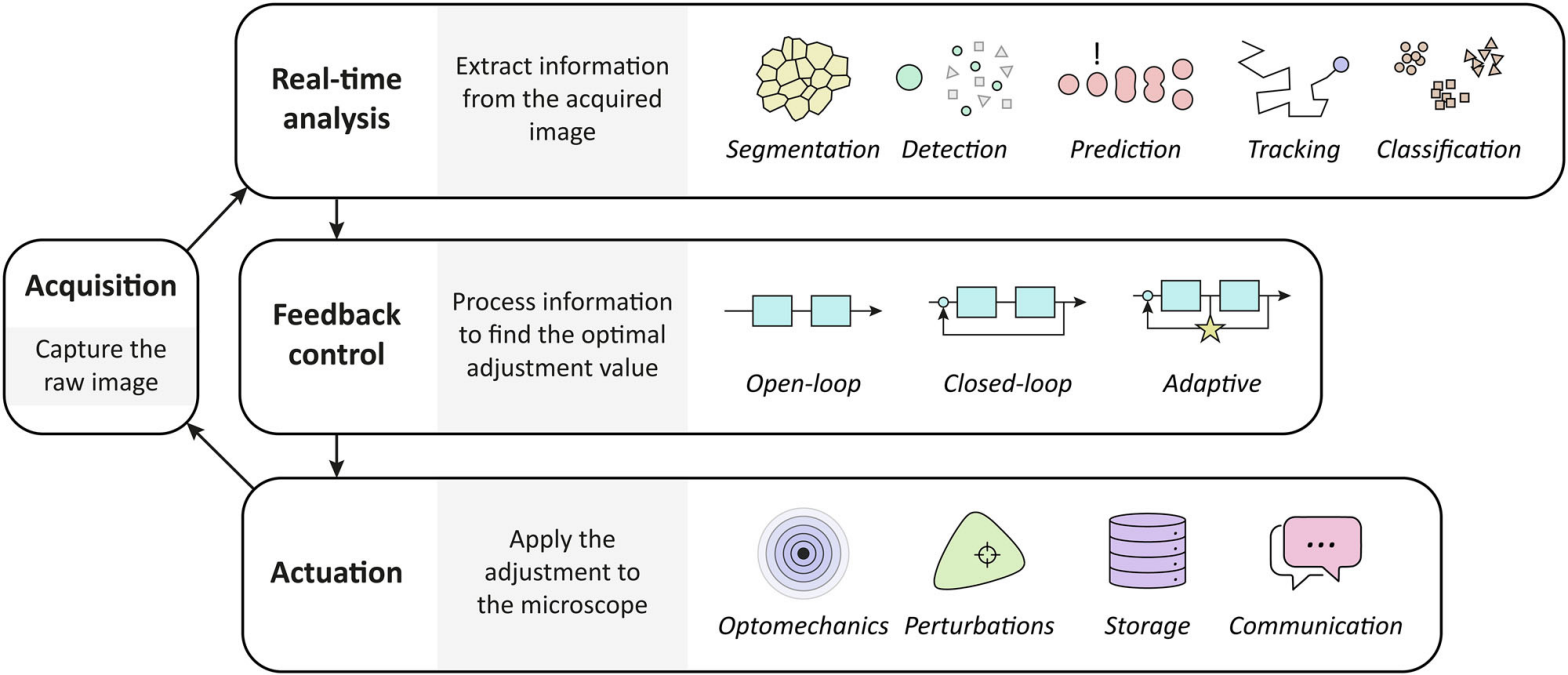
Strategies for achieving the defined goals of the smart microscopy experiment. After acquiring an image, information is extracted and fed into a control algorithm that is able to adjust an actuator. Over time, this results in optimized data collection for a given experiment.

Before delving deeper into the strategies, a differentiation between a priori and on-the-fly automation must be made. A priori automation means that the feedback loop to find the optimal experimental condition happens before the experiment itself. This automation is already well established in the field, and it is mainly used when the experimental conditions are not expected to change during the runtime of the experiment^133,134^. Conversely, on-the-fly automation means that the feedback loop happens simultaneously with the experiment, thus the conditions are continuously changed or actively maintained. Most of the new developments of smart microscopy focus on on-the-fly automation, therefore real-time analysis is required. An overview of the smart microscopy feedback loop is shown in Fig. 3.

### Real-time analysis

Which analysis is needed for a smart microscopy experiment strongly depends on the type of experiment. But in almost all cases, the main input for real-time analysis is the current image acquired by the microscope. From the current image, much information can be extracted^4^, such as morphology of cells or specimen, signal intensity, dynamic with respect to the previous frame, interaction with neighbor cells or specimen, etc. This information is then used by the microscope to make changes to the acquisition.

Some features of the sample can be readily visible to the human eye, yet remain challenging to extract or quantify automatically. Many image analysis tools tailored for biology help with extracting data from acquired images. Here, we break down common analysis strategies for segmentation, detection, tracking, prediction, and classification.

*Segmentation* is used to define the contour of an object of interest, either in 2D or 3D^135–137^. Commonly used to differentiate individual cells, segmentation algorithms give a mask of the object, which can then be used to study the morphology and position of the object of interest. Multiple segmentation algorithms exist in the literature, each of them with advantages and disadvantages. The most frequently used algorithms for cell and tissue are CellPose^138–140^, Segment Anything Model (SAM)^141–144^, and StarDist^145^. Furthermore, commercial solutions also exist, such as Imaris and Zeiss Arivis.

In many smart microscopy experiments, particularly for event-driven microscopy, a *detection* algorithm is needed to recognize the event that triggers the change in acquisition. What part of the signal is used for detection depends on the experiment, therefore the algorithm is highly experiment-dependent. Some commonly used signals include having a specialized fluorescence channel to detect intensity changes (either peaks or dips)^146,147^, comparing the morphology of the sample with a desired shape with algorithms such as template matching^148–150^, or recording the sample through time to detect changes in its dynamics (such as speed)^151–153^.

Instead of only detecting events, *prediction* algorithms can also be used to be ready for an event before this even happens. Prediction algorithms in machine learning are referred to as regression^154^, and with them it is possible to predict the future steps of a dynamic signal, such as movement or interaction of cells. Among regression algorithms, linear regression, decision trees, and random forest are the most common^155,156^. Furthermore, deep neural networks (NNs) are increasingly being used and show high accuracy for prediction^157–159^.

*Tracking* algorithms in microscopy are used with dynamic samples such as migratory cells and worms. Sometimes tracking is crucial to study the dynamics of the systems, other times tracking is used to ensure the dynamics of the system does not perturb the acquisition. If only the center position of the specimen is relevant, then tracking can be based on a reduced resolution of the image, treating it as a particle. Many algorithms for particle tracking exist in the literature^160,161^. If, instead, the shape of the specimen is relevant, segmentation along with tracking is needed^162^. Tools such as WormTracker and Deep-worm-tracker were developed specifically to track *C. elegans*^100,163,164^. Alternatively, for single-cell tracking many approaches exist, so many that in 2013 the cell tracking challenge was implemented^165–167^, to benchmark which approaches perform better for different scenarios.

Overlapping with regression are *classification* algorithms. When observing a population for example, classification and clustering help determine which group each individual belongs to^168,169^. Classification to label different objects of interest is particularly important for e.g. brightfield imaging where no fluorescence labels are present^170–173^. Classification can be done using algorithms such as K-Means and hierarchical clustering^174,175^. Nowadays, NNs are also widely used^176^. Although not classification algorithms, dimensionality reduction algorithms can be used to aid classification, such as Principal Component Analysis, Support Vector Machine, and Uniform Manifold Approximation and Projection^177–179^.

### Feedback control

Smart microscopy is possible only if the microscope can be controlled. In many smart microscopy experiments, the way the microscope is controlled is simple and does not require advanced algorithms. Examples of simple control strategies are binary control, such as turning on and off a light source sporadically, or built-in control, such as in newer mechanical stages where the device control is already optimized. For more complex experiments, however, choosing the correct control strategy is crucial.

Control theory is a well-established branch of engineering with broad applications. Control strategies are broadly classified into two categories: open loop and closed loop^180,181^. In both cases, the controller adds an input to the system to drive it to a certain state. The difference is that closed loop control takes into account the resulting state of the system, while open loop does not. Within closed loop control, an important subcategory is adaptive control. While common closed-loop control reacts consistently with fixed parameters, adaptive control uses modeling of the system to adapt the parameters on the fly to specific states to achieve better results. Importantly, adaptive control refers to the use of different control parameters based on the state of the system, not necessarily a change of the model on the fly to adapt to the system^181^.

These three types of control strategies, open loop, closed loop, and adaptive control, are applied in different types of smart microscopy experiments. *Open-loop control* is applied when control is only needed sporadically. Event-driven experiments, where the goal is to observe rare events, do not generally require feedback on the controller. An example of open-loop control in event-driven experiments is phototagging of cells of interest. If the phototagging process is well understood, once an event is detected the microscope automatically tags the corresponding cell with a predefined wavelength, power, and time.

*Closed-loop control* is predominantly used in experiments where the control input is regularly applied. This is normally the case for quality-driven and target-driven experiments, where the microscope is constantly changing its settings to maintain the quality of the image or the state of the sample. Here, having feedback on the current state of the system is crucial. In terms of control strategies, The proportional integrative derivative (PID) controller is widely regarded as the default solution in industry due to its simplicity and ease of implementation^182^. PID is composed of three components to minimize error, one focused on errors in the present, other on errors of the past, and the third one on the possible errors of the future. An example of closed-loop control in target-driven experiments is depth tracking of single particles^183^. In techniques such as single-molecule spectroscopy, maintaining the focus on a single particle is key. Some experiments use closed-loop real-time control to keep track of the depth of the particle and ensure the single molecule is in focus throughout the experiment.

Closed-loop control strategies have been applied to biology in the past, particularly in synthetic biology to control biological processes at a larger scale^184–186^. Typical biological applications and experiments are challenging for classical control strategies due to its stochasticity, but more importantly, heterogeneity^187^. Thus, if the goal of the smart microscopy experiment is to control the biology, such as in outcome-driven experiments, *adaptive control* is often required^132^. Adaptive control can be based on analytical models or, more recently, on machine learning techniques and AI models^188,189^. AI tools based on deep learning, reinforcement learning, adversarial or one-shot learning, where artificial NNs learn the underlying behavior of the sample and can compensate and adapt on the fly for variations in dynamics or noise, show promise for applications in smart microscopy^59^. Using advanced control algorithms with model-based inference or AI not only allows us to control the observed biological processes with higher precision, but also opens the door to extract information from the biology by observing how it reacts to the control input^190^. Quality-driven microscopy can use the control performance to infer the health or condition of the sample, while outcome-driven microscopy, thanks to its controlled perturbations, can use the control information to develop a dynamic model of the sample.

### Actuation

Here, we discuss the set of available devices that can be adjusted in a smart microscopy experiment, driven by the feedback controller. To help guide the researcher to think about how they might wish to implement it in their research, actuators are separated into four categories depending on what they control: optomechanics, perturbations, storage, and communication. As defined earlier, a smart microscope modifies the acquisition parameters on the fly. Thus, we consider any on-the-fly control of one or more of the actuators described below as a change in acquisition parameters and thus can be classified as smart microscopy.

How smart microscopy is implemented strictly depends on the type of microscope and imaging modality being used. Therefore, when designing a smart microscope, it is crucial to consider what components are available for optimization and what is needed for each specific imaging modality. To help the reader in this process, we grouped commonly-used controllable parameters by microscopy technique. This information is shown in Table 1. The information presented also helps the user to select a suitable microscopy modality or to adapt an existing model to allow for a specific automation experiment.

**Table 1:** Microscopy techniques and examples of their optimizable feedback parameters.

Modality	On-the-fly optimisable parameter	Smart Microscopy Strategy groups
Universal for multiple microscopy modalities	Multidimensional acquisition control	Optomechanics
	Camera parameters (exposure, binning, gain…)	Optomechanics
	Shutter control	Optomechanics
	Stage movement	Optomechanics
	Tiling and positions	Optomechanics
	Drift correction	Optomechanics
	Z-stack range/step	Optomechanics
	Focus	Optomechanics
	Illumination wavelength	Optomechanics Perturbations
	Illumination intensity	Optomechanics
	Photomanipulation	Optomechanics Perturbations
	Objective selection	Optomechanics
	Excitation/emission filter sets	Optomechanics
	Adaptive optics	Optomechanics
	Environmental control (temperature, CO₂…)	Perturbations
	Perfusion chambers	Optomechanics Perturbations
	Autosave settings	Storage
	Light path	Optomechanics
	Real-time data processing	Storage Communication
Specific for brightfield microscopy	Contrast enhancement	Optomechanics
	Condenser alignment and aperture	Optomechanics
Specific for confocal (laser scanning) microscopy/STED/MINFLUX	Detector wavelength range	Optomechanics
	Line/frame averaging	Optomechanics
	Scan speed	Optomechanics
	Scan region	Optomechanics
	Laser beam shape	Optomechanics
	Pinhole size	Optomechanics
Specific for Total Internal Reflection Microscopy (TIRF)	Incident angle	Optomechanics
Specific for Structured Illumination Microscopy (SIM)	Pattern structure	Optomechanics
	Pattern frequency	Optomechanics
	Phase shifts	Optomechanics
Specific for Light-Sheet Microscopy	Light sheet thickness (waist)	Optomechanics
	Light sheet angle	Optomechanics
	Sample rotation	Optomechanics
Fluorescence Lifetime Imaging Microscopy (FLIM)	Time-gating settings	Optomechanics

*Optomechanics* involves most motorized components of a microscope, such as stage movement, depth position, objective selection, filter wheel selection, and imaging area. Optomechanics is also used to control light intensity, wavelength, wavefront correction, and more^2,191^. Optomechanics also refer, when applicable, to external components such as motorized arms, e.g., in high-throughput systems.

Optomechanics actuators can be used, for example, to dynamically change the FOV^192^ by moving the stage (e.g., when using brightfield) or changing the scan region (e.g., when using laser-scanning confocal). Another example is to dynamically change the imaging wavelength^193,194^ (both excitation and detection) by changing excitation and emission filter sets (e.g., in epifluorescence) or by changing laser wavelength and spectral detection range (e.g., in confocal).

*Perturbations* can be any type of induced signal that induces changes in the sample, such as light, flow-in of a drug, heat, pressure, etc^195,196^. These are automated, controlled inputs into the sample, such that the sample reacts to it in some manner. Perturbations are generally applied to observe the reaction of the sample, but it can also be used to maintain conditions rather than changing them.

A key factor of perturbations described here is that they are applied voluntarily. There are many involuntary factors that may affect the sample under study that are either unavoidable (e.g., phototoxicity) or a by-product of another effect (e.g., heat due to illumination). Such effects are not considered as a voluntary and controlled perturbation. Furthermore, a single perturbation can have multiple effects depending on the experiment and the way it is applied. For example, a localized light input can be used for optogenetics, photoactivation, or FRAP^197,198^. As highlighted in Table 1, different parameters can be adjusted for perturbation control, such as environmental control^199^ (temperature and CO_2_) or illumination power and wavelength. Perturbations are linked to outcome-driven experiments, as such experiments need an external perturbation to control the biology. However, perturbations can be present in experiments with other goals as well. An event-driven experiment, for example, can use a perturbation such as photoactivation or fixation after finding an event.

*Storage* can be seen as a virtual actuator with which storage and data handling are automated. This includes data compression, cropping, data deletion, etc^200,201^. An example where smart data management is beneficial is event-driven light sheet microscopy. When the data prior to the event or far from the location of the event is irrelevant, deleting or compressing that data on the fly is crucial to reduce storage and allow long-term acquisitions.

For storage-based automation to be considered smart microscopy it must be an automation needed during the experiment. When data analyses are carried out after the experiment is finished, then they are completely decoupled from the experiment itself. Instead, if storage-based automation is done before the experiment (e.g., to define formatting and memory allocation) or on the fly (e.g., on-the-fly image deskewing) the experiment is then fundamentally changed thanks to the automation^80^. On-the-fly data handling will require dynamic metadata formats, especially if the data is to be compared with experiments from other devices. Table 1 includes real-time processing as an adjustable parameter to have control over data storage.

Finally, *communication* is another virtual actuator which connects the microscope and the user (or other machines)^202^, providing or receiving information about the current experiment in an user-friendly manner. The communication could be prior to the experiment to define the experiment parameters or during the experiment to notify the user or to wait for user action. This may include graphical interfaces, where the user selects areas of interest, or data upload of example images for training, or web-based data transfer and notifications. Table 1 shows the adjustable characteristic of the microscope that can trigger external communication as real-time processing.

Communication-based automation is drawing a lot of attention with the surge of LLMs such as chatGPT. Discussions in the community about involving LLMs in the experiment design soon emerged^203,204^. Being able to communicate with the microscope in plain language would be particularly beneficial for non-experts and for teaching microscopy and may alleviate the load of experiment design.

## Current challenges

Despite the significant advancements in smart microscopy, several challenges and limitations remain. One key issue is the balance between user-bias and algorithm-bias. While smart microscopy systems aim to reduce human intervention, there is still a risk of user biases influencing the settings, analysis, and interpretation of results, particularly in the design and application of machine learning algorithms. Conversely, algorithms themselves may introduce their own biases based on the data they were trained on, leading to inaccurate or incomplete interpretations of biological phenomena. The so-called explainable AI (xAI)^205^, where the reasoning of AI algorithms is described to the user, may help to make them aware of the biases.

Even though many popular AI algorithms only need limited fine-tuning, there is often still a requirement for well-annotated dataset, something that can limit the reproducibility. Furthermore, the “black box” nature of many AI algorithms undermines the transparency and reproducibility. The future advancement of smart microscopy will depend not merely on algorithmic improvements but also on developing standardized protocols, metadata conventions, expanding open-access resources, and creating more intuitive interfaces that democratize access to these methods.

Another challenge lies in interoperability. Many smart microscopy systems involve a variety of software, hardware, and imaging modalities, often from different manufacturers. Nowadays, some microscope manufacturers also offer their proprietary automation and smart microscopy solutions^63^. Ensuring that these systems can seamlessly work together and share data remains a significant hurdle, as compatibility issues can disrupt workflows and limit the utility of integrated systems. Open-access application programming interfaces (APIs) with universal protocols will be needed in the future to ensure most software can operate across most hardware. The challenges of interoperability and the combination of both proprietary and open access platforms is addressed in the white paper of the Smart Microscopy Working Group from Euro-Bioimaging^63^.

Data handling and formatting is another critical area that requires attention, particularly as the volume and complexity of data generated by smart microscopes increase^206^. The massive data volumes generated by high-resolution, time-lapse imaging create substantial challenges in storage, management, and processing, necessitating robust IT infrastructure. Reproducibility across different systems is further hampered by the absence of standardized calibration and acquisition protocols. The future of smart microscopy will likely involve smart data management solutions that can handle large datasets, ensuring they are efficiently processed, filtered, stored, and analyzed. Such a pipeline furthermore requires accessible file formats and metadata. This will require developments of AI-driven tools capable of providing meaningful insights in real-time, particularly to understand what data is relevant to keep. Indeed, there are current initiatives that aim to develop such robust file format standardization, such as the Open Microscopy Environment^201^.

The complexity of smart microscopy systems is also a limitation. There is no “one-size-fits-all” solution, as different biological specimens and experimental goals require tailored approaches. The diversity of microscopy techniques and the need for highly specific configurations mean that users often face a steep learning curve and must customize systems to their particular needs. For this reason, it is important to develop modular and system-agnostic solutions from the conception, so techniques can be combined or anatomized to be reused in different experiments. Furthermore, universal smart microscopy solutions face the challenge of heterogeneity in biology. The large variability in biological samples needs to be accounted for when designing automated experiments.

Finally, making these technologies easily accessible for biologists and other users is another ongoing challenge. While smart microscopy offers powerful tools, they often require specialized knowledge and expertise to operate effectively. This increases the entry barrier and will delay the adoption of smart microscopy solutions in biology, where alternative solutions were developed in the past trying to tackle the problems now smart microscopy can solve. Simplifying user interfaces, enhancing automation, and providing user-friendly training resources will be critical for increasing accessibility, ensuring that these systems can be effectively used by biologists without extensive technical backgrounds.

These challenges highlight the need for continued innovation and collaboration across hardware, software, and user experience design to make smart microscopy more effective, accessible, and widely adopted in biological research.

## SmartMicroscopy.org

The history and development of smart microscopy has shown that community-driven approaches are essential for the progress of the field. Smart microscopy lies at the intersection of many research fields and collaboration and sharing of knowledge is the most effective way to develop new techniques and tools. To facilitate this continued collaboration, we set up the website smartmicroscopy.org for crowd-sourcing of information regarding smart microscopy.

The website contains four types of content: education, literature, resources, and news. Education encapsulates the information presented in this review, with the history and classifications for new readers. Literature includes the references of this review, with a search engine and filters, and the advantage to be dynamic, such that new publications can be added. Resources include programming repositories and commercial tools for microscope control and analysis. And finally, we collect news from the field such as upcoming events, new software releases, and press releases of scientific articles.

Visitors can contact us to add new literature, resources, or news. For literature and repositories, this is as simple as using the available forms to send a DOI or URL linked to the publication or code repository. The website smartmicroscopy.org is a community partner of forum.image.sc, an online forum that members can use for discussions related to smart microscopy. Furthermore, we actively collaborate with the euro-bioImaging SMWG which hosts the github-based website smartmicroscopy.github.io, gathering information regarding interoperability in smart microscopy and how to combine academic and industry efforts. The literature presented on the website smartmicroscopy.org is connected to a Zotero library that is publicly accessible. The website is intended to be community built, so we encourage people to propose changes, corrections, or new information.

## Conclusions

With this review, we present an intuitive classification of smart microscopy with highlighted representative examples, as a guide for researchers to use and optimize smart experiments. We hope that our classification and our introductory description of smart microscopy, along with key examples of it, will help new readers to get familiar with the concepts and we regret any omissions due to space constraints. Finally, as smart microscopy is a highly interdisciplinary research area, we look forward to a future where scientists from various disciplines contribute their knowledge to this rapidly evolving field.

## Data Availability

No datasets were generated or analyzed during the current study.
